# Supplementary material for: Identification of Sex-Specific Markers and Candidate Genes Using WGS Sequencing Reveals a ZW-Type Sex-Determination System in the Chinese Soft-Shell Turtle (Pelodiscus sinensis)
Source: Int J Mol Sci. 2024 Jan 9;25(2):819. doi: 10.3390/ijms25020819 (PMC10815769; doi:10.3390/ijms25020819)
Supplement: Supplementary file 1 [file ijms-25-00819-s001.zip › Figure S1. PCR validation of female and male specific sequences in P. sinensis.pdf]

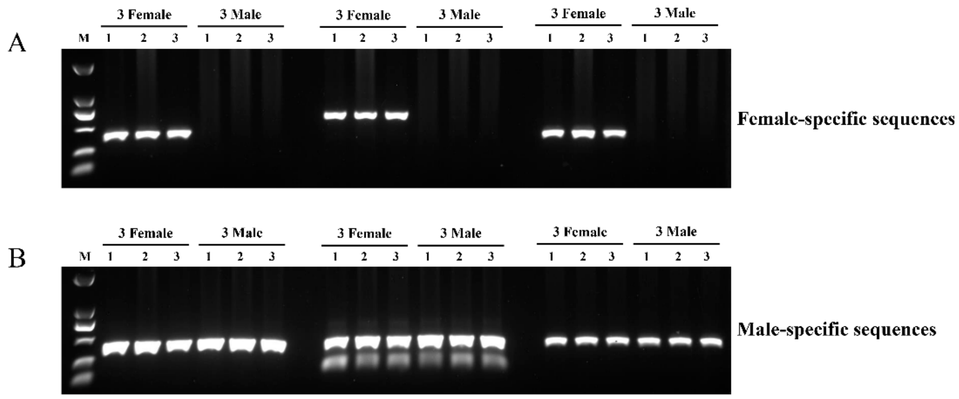

Figure S1. PCR validation of female (A) and male (B) specific sequences in *P. sinensis*. Based on the female and male specific sequences obtained from the first round of screening, three pair primers were designed to verify the authenticity of the sex-specific sequences in three females and three males, and to preliminary evaluate of the sex determination system of the sequenced species, respectively. M, DL 2000 DNA marker.
